# Exploring Functioning Impairment in Clinical Course of Illness in Early and Mid-Life Onset Bipolar Disorder in Mexican Patients

**DOI:** 10.1192/j.eurpsy.2025.1059

**Published:** 2025-08-26

**Authors:** H. L. Juarez, A. B. Cuellar, S. Luna, M. S. Gonzalez

**Affiliations:** 1 Psychiatry, Universidad Autonoma de Nuevo Leon, Monterrey, Mexico; 2 Psychiatry, Mayo Clinic, Rochester, United States

## Abstract

**Introduction:**

Bipolar disorder (BD) is a chronic illness that can lead to severe functioning impairment, which can disrupt daily life. This study aims to compare overall functioning and clinical presentation associated with early onset (EO) and mid-life onset (MO) BD.

**Objectives:**

The objective of this study is to be able to predict the cognitive and functional impairment evolution of bipolar disorder based on the onset.

**Methods:**

A total of 154 BD patients (81 in euthymia and 51 in a depressive episode) participated in this cross-sectional study. We selected two groups of subjects based on age at onset of the first mood episode (EO: 0-20 years, n=117; MO: 20-40 years, n=37) and compared socio-demographic, clinical variables and functional impairment assessed by the World Health Organization Disability Assessment Schedule (WHODAS 2.0).

**Results:**

75.2% of the patients with an EO were female, they had higher psychiatric comorbidities prevalence (76.1% vs. 43.2%; p<.001), being personality disorders the most significant (30.2% vs. 5.4%; p=.001), more suicide attempts (51.3% vs. 16.2%; p<.001), mood episodes (mdn=12, iqr=17 vs. mdn=6, iqr=9.5; p=.001), childhood abuse (93.5% vs. 73.9%; p=.014), being verbal (78.0% vs. 52.2%; p=.015) and physical abuse (51.6% vs. 21.7%; p=.009) higher compared to MO. Functionality was also affected, with higher dysfunction percentages in EO (mdn=47, iqr=34.7 vs. mdn=24, iqr=12.5; p<.001) affecting cognition (mdn=8.5, iqr=6 vs. mdn=4, iqr=7; p<.001), self-care (mdn=2, iqr=5 vs. mdn=0, iqr=1; p<.001), daily activities (mdn=12, iqr=13 vs. mdn=4, iqr=10; p<.001) and community participation (mdn=13, iqr=8.7 vs. mdn=8, iqr=7; p<.001).

EO depressed patients (n=38; F: 30; M: 8), had more personality disorders (42.1% vs. 0%; p=.010), suicide attempts (60.5% vs. 0%; p<.001), mood episodes (mdn=12, iqr=12 vs. mdn=5.5, iqr=4.7; p=.001), lower overall functionality (mdn=57, iqr=41.5 vs. mdn=35, iqr=38.3; p=.009), cognition (mdn=10, iqr=6.5 vs. mdn=4, iqr=9; p=.023), self-care (mdn=4, iqr=6.2 vs. mdn=0, iqr=3; p=.008), oneself care (mdn=9, iqr =7 vs. mdn=5, iqr=8.5; p=.025), daily activities (mdn=15.5, iqr=14 vs. mdn=11, iqr=12.7; p=.037) and community participation (mdn= 15.5, iqr=8.2 vs. mdn=11.5, iqr=5.7; p=.030).

EO euthymic patients (n=59; F: 42; M: 17) had more psychiatric comorbidities (74.6% vs. 33.3%; p=.001), physical abuse history (51.2% vs. 0%; p= .002), and lower functionality (mdn=7, iqr=7 vs. mdn=2, iqr=7; p=.004), cognition (mdn=7.5, iqr=7 vs. mdn=4, iqr=7; p=.033), daily activities (mdn=9, iqr=11 vs. mdn=3, iqr=8.5; p=.005) and community participation (mdn=10, iqr=9 vs. mdn= 7, iqr=7; p=.031).

**Conclusions:**

Results suggest that patients with an EO are more associated with severe psychosocial functioning impairment. Future studies are needed to clarify a more severe illness prediction between EO and MO.

**Disclosure of Interest:**

None Declared

